# The Impact of Calcium Depletion on Proliferation of *Chlorella sorokiniana* Strain DSCG150

**DOI:** 10.4014/jmb.2403.03018

**Published:** 2024-05-29

**Authors:** Soontae Kang, Seungchan Cho, Danhee Jeong, Urim Kim, Jeongsug Kim, Sangmuk Lee, Yuchul Jung

**Affiliations:** Daesang Cellgene Corporation, Yongin-si 17095, Republic of Korea

**Keywords:** Ca^2+^, *Chlorella sorokiniana*, growth, cell cycle, gene expression, fission

## Abstract

This study analyzed the effects of Ca^2+^ metal ions among culture medium components on the *Chlorella sorokiniana* strain DSCG150 strain cell growth. The *C. sorokiniana* strain DSCG150 grew based on a multiple fission cell cycle and growth became stagnant in the absence of metal ions in the medium, particularly Ca^2+^. Flow cytometry and confocal microscopic image analysis results showed that in the absence of Ca^2+^, cell growth became stagnant as the cells accumulated into four autospores and could not transform into daughter cells. Genetic analysis showed that the absence of Ca^2+^ caused upregulation of calmodulin (*calA*) and cell division control protein 2 (*CDC2_1*) genes, and downregulation of origin of replication complex subunit 6 (*ORC6*) and dual specificity protein phosphatase CDC14A (*CDC14A*) genes. Analysis of gene expression patterns by qRT-PCR showed that the absence of Ca^2+^ did not affect cell cycle progression up to 4n autospore, but it inhibited *Chlorella* cell fission (liberation of autospores). The addition of Ca^2+^ to cells cultivated in the absence of Ca^2+^ resulted in an increase in n cell population, leading to the resumption of *C. sorokiniana* growth. These findings suggest that Ca^2+^ plays a crucial role in the fission process in *Chlorella*.

## Introduction

*Chlorella* is a photosynthetic microalga that is widely used as an ingredient in foods, dietary supplements, and animal feedstock. Various culture media containing trace metals have been developed and are being used for commercial production of *Chlorella*, but the biological effects of each medium component have not been clearly identified. While the types and concentrations of trace metals required may vary depending on the microalgal species, they are essential materials for growth. Metallic elements affect microalgal growth by activating enzymes involved in substrate metabolism, as well as by being involved in various processes, such as DNA synthesis, cell division, and maintaining the ion balance and cell membrane. To determine the effects of metallic elements in growth medium on *Chlorella* cell growth, we observed *Chlorella* cell growth in the absence of metallic elements in growth medium and confirmed that of the metallic elements, Ca^2+^ has the most critical role in cell growth (Fig. S1).

Generally, Ca^2+^ acts as a secondary messenger in various cellular processes, including cell proliferation, development, motility, secretion, learning, and memory, thereby influencing nutrient assimilation, growth, and stress adaptation in microalgae and plants [1]. It has been observed that Ca^2+^ enhances the uptake of nitrogen, organic carbon sources and phosphorus, leading to improved growth rates in *Chlorella pyrenoidosa* [2] and *Scenedesmus* sp. [3]. Moreover, in *Chlorella vulgaris*, higher concentrations of Ca^2+^ increased growth rate and enhanced resistance to reactive oxygen species generated by gamma irradiance [4]. It has also been reported that Ca^2+^ starvation results in the reduction of biomass yield in *C. vulgaris* [5]. Growth of subterranean clover plants has been reported to be stunted by calcium deficiency [6]. Calcium deprivation in *Spinacia loeracea* increased superoxide radicals and decreased the activities of antioxidant enzymes, resulting in growth inhibition of the spinach [7].

Calmodulin (CaM), the primary intercellular receptor of Ca^2+^, plays an important role in regulating cellular responses. Serine/threonine phosphatase calcineurin and Ca^2+^/CaM- dependent protein kinases (CaMKs) are key Ca^2+^ target proteins that play a central role in the mammalian cell cycle transition and regulation, while deficiency of these proteins can inhibit cyclin D1/Cdk4 activation causing early/mid G_1_ arrest and promotes p21/p27 accumulation, leading to late G_1_ or S phase arrest in the cell cycle [8-11]. In mammalian and fungal systems, CaMKII is a target protein of Ca^2+^/CaM involved in G_2_ to M and anaphase to metaphase transitions, and inactivation of Cdc2 by deficiency and inhibition of CaMKII induces G_2_ phase arrest [12-18]. Thus, the importance of Ca^2+^/CaM in eukaryotic cell proliferation has been conclusively established, however, its role in *Chlorella* cell proliferation remains unknown.

*Chlorella* are maintained in haploid form and produce asexually reproducing autospores by mitosis [19, 20]. Reproduction typically involves the production of four daughter cells within a mother cell, and when these daughter cells become mature, the cell wall of the mother cell ruptures to enable the daughter cells to divide and emerge [21].

In this study, we used flow cytometry and confocal image analysis to investigate the effects of Ca^2+^ on the proliferation and cell cycle of *C. sorokiniana* strain DSCG150 and analyzed genetic expression to identify the characteristics of downstream target gene expression according to the presence/absence of Ca^2+^ supply.

## Material and Methods

### Preparation of Experimental Strain and Medium Composition

The strain used in the experiment was a pure isolate of the *C. sorokiniana* strain DSCG150 (NCBI accession number: JAXCUZ000000000). The culture medium used was Sanawa and Endo medium IV [22], which was modified with 20 g/l of glucose and 5 g/l of urea for optimal cultivation of DSCG150, while the solid medium was prepared by adding 20 g/l of agar (Samchun, Republic of Korea). Cells were streaked on fresh solid medium every 5 days and incubated at 30°C incubator under dark condition.

### *C. sorokiniana* Strain DSCG150 Growth according to Presence/Absence of Ca^2+^ Supply

*C. sorokiniana* strain DSCG150 was grown in different concentrations of Ca^2+^: 0.5, 1.0, and 2 mM. DSCG150 growth was the same across all Ca^2+^ concentrations, therefore, the lowest concentration (0.5 mM) was selected for the subsequent experiment (Fig. S2).

For observation of changes in cell growth, three different Ca^2+^ supply conditions were applied.

The three conditions were divided into 1) 0.5 mM of Ca^2+^ added (normal growth, NG); 2) without Ca^2+^ added (Ca^2+^ absence, CA); and 3) cultivation without Ca^2+^, but 0.5 mM of Ca^2+^ added after 48 h (Ca^2+^ replenishment, CR). *Chlorella* cultivated for 5 days on the solid medium was diluted in sterile distilled water and centrifuged at approximately 4,000 ×*g* for 10 min (Avanti J-E, Beckman Coulter, USA). Cells were washed twice by removing the supernatant and resuspending the cell pellet in sterile distilled water. The prepared cell suspension was inoculated to 250 ml of culture medium in a 1L baffled Erlenmeyer flask (DU.2128354, DURAN, Germany) to reach a final optical density of 0.4–0.45 (OD at 600 nm; OD600). The medium composition was the same as described in Section 2.1., with the addition of CaCl_2_·2H_2_O in the medium, according to the Ca^2+^ supply conditions. The cells were cultivated at 30°C with shaking at 140RPM under dark conditions in an ISS-7200R shaking incubator (JEIOTECH, Republic of Korea). During cultivation, samples were taken every 4 h and OD600 was measured using a 96-well microplate reader (BioTek Instruments, Inc, USA). Subsequently, cells were counted using Neubauer chamber (Paul Marienfeld GmbH & Co. KG, Germany). The residual glucose and Ca^2+^ concentration in the sample solution were measured using a Cedex Bio Analyzer (Roche, Swiss).

### Flow Cytometry Analysis

To analyze the pattern of dividing cell nuclei of *C. sorokiniana* strain DSCG150, the cells were stained with propidium iodide (PI)/RNase (BD Biosciences, USA) and analyzed using flow cytometry (FACSMelody, BD Biosciences). For PI staining, 100 μl of sample solution was treated in 100°C water for 10 min, followed by centrifugation at 3,500 RPM for 2 min and staining of the pellets with 100 μl of PI for 10 min at room temperature. For flow cytometry analysis, the stained cells were centrifuged at 3,500 RPM for 2 min, after which, the pellets were suspended in 500 ul of PBS. After doublet discrimination by forward and side scatter, single cells were counted at 10,000 cells per sample and detected by PE (YG) and APC-Cy7 channel lasers.

### Confocal Microscopic Analysis

The sample solution (100 μl) was centrifuged at 3,500 RPM for 2 min. The supernatant was discarded and, the pellets were stained in 100 μl of Hoechst 33342 (Thermo Fisher Scientific, USA) at 37°C for 10 min. After applying the sample on slides and fluorescence was observed at an excitation wavelength of 352 nm and an emission wavelength at 454 nm for Hoechst 33342, and an excitation wavelength of 680 nm and an emission wavelength of 697 nm for Atto 680 using a Stellaris 5 (Leica, Germany). Moreover, the quantum yield and lifetime of fluorescent materials were used for detection on only the autofluorescence region of chlorophyll through Leica TauSense which can filter color development by fluorescent dye.

### RNA Sequencing and Differential Expressed Gene Analysis

To observe the gene expression patterns of *C. sorokiniana* strain DSCG150 according to the presence/absence of Ca^2+^ in the medium, early log-phase (32 h) samples were obtained under CA and NG conditions. Total RNA extraction was performed using Hybrid-R 100p (GeneAll, Republic of Korea), in accordance with the manufacturer’s instructions. The extracted RNA was sent to Macrogen (Republic of Korea) to create a library, followed by paired-end 100 bp sequencing using NovaSeq6000 (Illumina, USA). The total reads obtained were mapped on a reference genome, the whole genome sequence contig of *C. sorokiniana* strain DSCG150 (NCBI accession number: JAXCUZ000000000), information on aligned reads was used to perform transcript assembly and the expression levels of transcripts of each sample were analyzed. Differential expressed genes (DEGs) were annotated using databases available from GO, InterPro, PFAM, CDD, TIGRFAM, and Eggnog. Genes that showed significant differences in expression (|log2 FC|> 1 (FDR< 0.05)) under CA condition, relative to NG condition, were selected. The normalization of the expression level of transcripts was conducted using the trimmed mean of M-values (TMM) by edgeR program. To check the differences in expression of the selected genes according to the cell growth phase, intracellular gene expression at 24, 32, and 44 h based on lag, early-log, and log phases under NG culture condition was analyzed using qRT-PCR.

RNA was extracted from cells at each growth phase and synthesized into cDNA (RevertAid H minus First Strand cDNA Synthesis Kit, Thermo Fisher Scientific). qRT-PCR was performed using QuantStudio5 (Thermo Fisher Scientific) on the selected genes (Primer pairs, Table S1). The ΔΔCt method was used to analyze the relative quantification.

### Effects of Elimination of Metal Ion Source on Growth of *C. sorokiniana* strain DSCG150

DSCG150 growth depending on the presence or absence of trace metals in the culture medium was analyzed using Bioreactor RTS-8 (BIOSAN, Latvia). Sanawa and Endo medium IV was used as the basic medium and media with individual elimination of metal ions, except Mg^2+^, were used. The metal ions eliminated from the medium were Ca^2+^, Cu^2+^, Zn^2+^, Fe^2+^, Mn^2+^, Mo^2+^, and B^3+^. Cells cultivated in solid medium for 5 days were washed and inoculated into 50- mL BIOSAN tubes containing 20 ml of each medium to reach an initial OD600 of 0.6. During incubation under conditions of 30°C, 2700 RPM (kLa of 450/h), growth was observed by measuring OD600 every 6 h.

## Results

### Ca^2+^ Affected Cell Growth of *C. sorokiniana* strain DSCG150

**Fig. 1:**
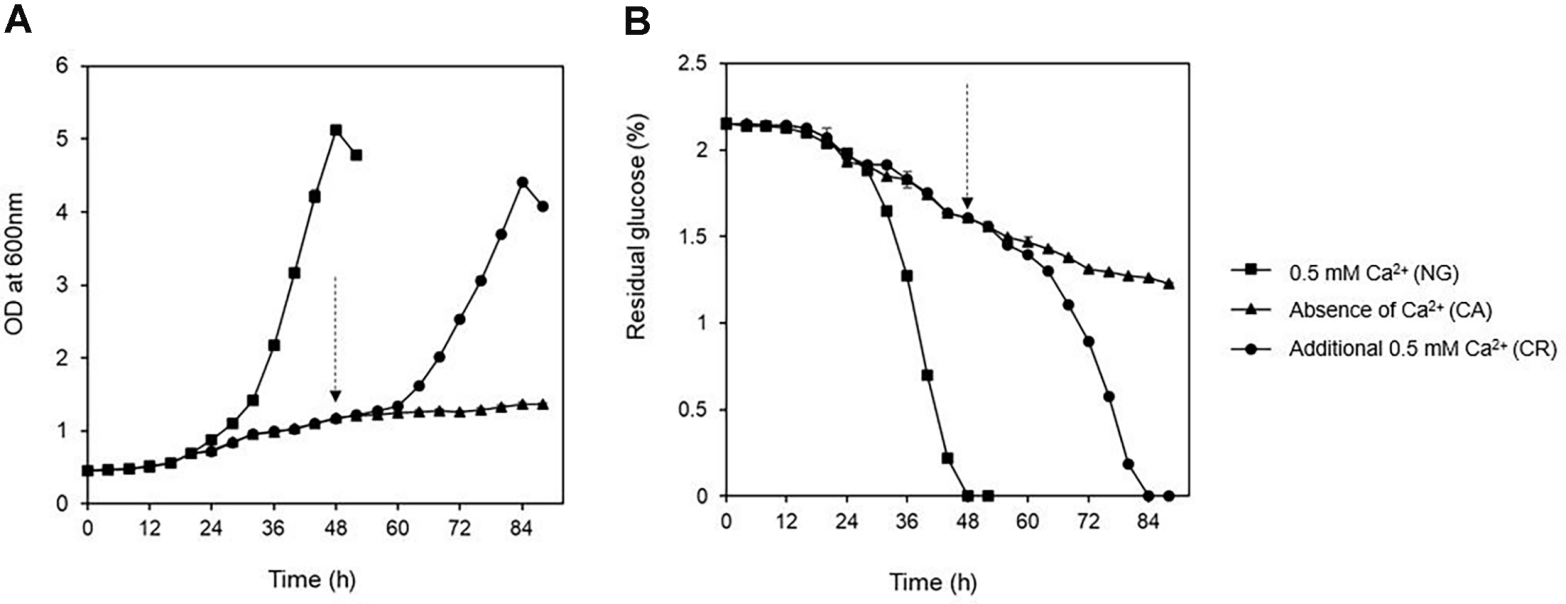
Biomass growth (A) and substrates consumption (B) of *C. sorokiniana* strain DSCG150 in the medium with 0.5 mM Ca^2+^ (NG), absence of Ca^2+^ (CA) and additional 0.5 mM Ca^2+^ (RS). The arrows represent a point to supply an additional 0.5 mM Ca^2+^. The values are shown as mean ±SD from three independent replicates.

The effects of Ca^2+^ on *Chlorella* growth over time were observed under varying conditions. DSCG150 showed an exponential growth with a specific growth rate of 0.073/h under NG condition, whereas under the CA condition, exponential growth was not observed (Fig. 1). Under NG condition, at 48h (the time point at which all glucose in the medium was consumed), OD600 of ≥ 5.0 and cell numbers of 5.01 ± 0.08 × 10^8^ cells/ml were observed. Under the CA condition, at 48 h, the mean glucose consumption was 7.7 g/l, OD600 was approximately 1.0, and the cell count of 0.61 ± 0.01 × 10^8^ cells/ml, indicating that growth was considerably inhibited. Under CR condition, *Chlorella* consumed all glucose (20 g/l) within 36 h of Ca^2+^ replenishment with a growth rate of 0.051/h, OD600 of 4.4, and cell numbers of 4.90 ± 0.05 × 10^8^ cells/ml (Fig. 1A). Under each culture condition, the amount of Ca^2+^ consumed was 1.14 mg in 48 h under the NG condition and 1.90 mg in 36 h under the CR condition. The findings confirm that Ca^2+^ is a key limiting factor for cell growth (Table 1).

### Ca^2+^ Led to Cell Proliferation of *C. sorokiniana* Strain DSCG150

**Fig. 2:**
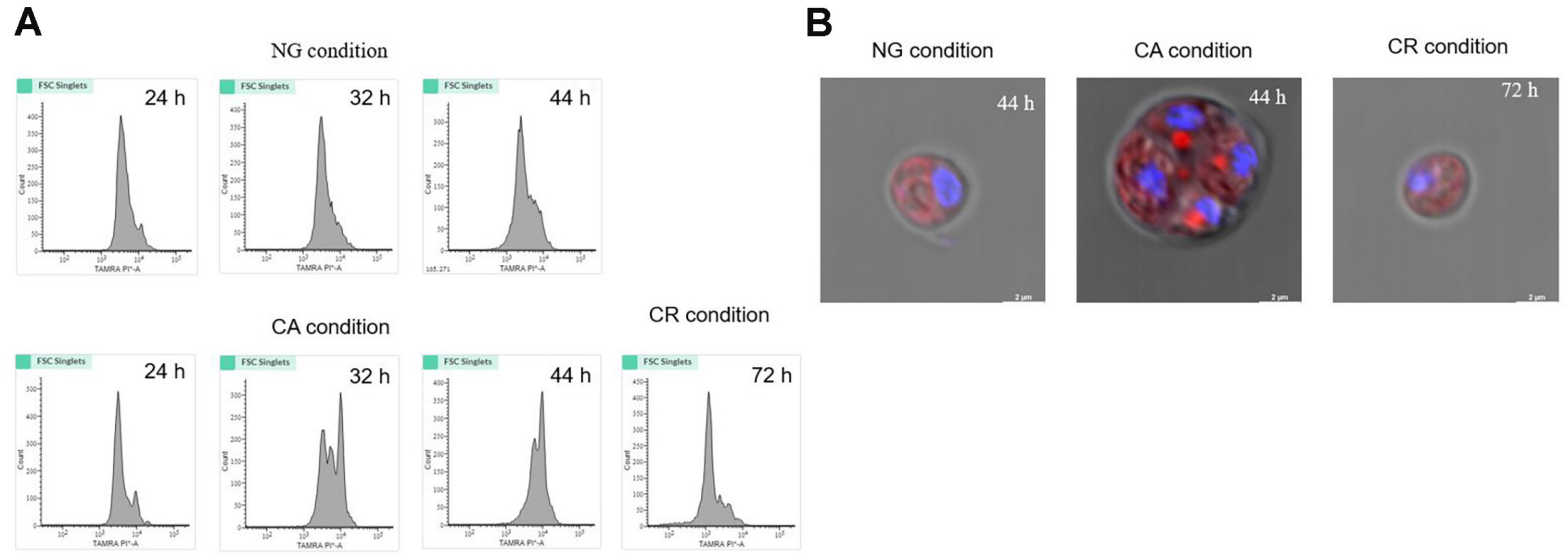
Flow cytometry analysis of cell proliferation (A) and confocal microscopic images (B) of *C. sorokiniana* strain DSCG150 in the medium with 0.5 mM Ca^2+^ (NG), absence of Ca^2+^ (CA), and additional 0.5 mM Ca^2+^ (CR). The additional 0.5 mM Ca^2+^ was inoculated after 48 h into culture broth of CA condition. Cells at each growth phase were thermal fixed and stained by PI dye for the flow cytometry analysis. X- and y-axes represent fluorescence intensity and cell number, respectively. For confocal microscopic analysis, live cells from each growth phase were stained with 20 mM Hoechst 33342. In confocal microscopic images, merged fluorescence images were shown, area emitting blue-cyan. fluorescence shows binding the dye to dsDNA minor grooves in a single cell. Red area shows autofluorescence of the corresponding cell. 24 h, cells in lag-phase; 32 h, in early log-phase; 44 h, in log-phase; 72 h, in log-phase. The growth phase was defined based on the growth curve in NG and CR conditions due to no logarithmic growth occurring in the CA condition.

For observation of cell proliferation, cells were treated with PI, a DNA intercalating agent, and analyzed using flow cytometry. The histogram of PI-stained DSCG150 showed a multimodal distribution with four intense peaks. As cells divided, the fluorescence intensity of the peaks for n, 2n, 4n, and 8n cells was analyzed to be 3 × 10^3^, 6 × 10^3^, 1.0 × 10^4^, and 2.0 × 10^4^, respectively (Fig. 2A). Under the NG condition, the n and 4n populations consisted of 64–69% and 8–14% of cells, respectively. Under the CA condition, a similar cell distribution was observed up until 24 h, however the intense fluorescence peaks of 2n and 4n increased by approximately 27% and 31%, respectively, at 32 h. At 44 h, fluorescence intensity at 4n increased by 41%. The cell distribution under the CR condition was comparable to that under the CA condition, but with Ca^2+^ replenishment at 48 h of incubation, the cells showed accumulation of n cells, similar to the pattern of fluorescence peaks indicating logarithmic growth under the NG condition.

Confocal microscopy analysis revealed that under the NG condition, the n cell population was the largest. Under the CA condition, during the early stages of cultivation, the n cell population was predominant, as cultivation time increased two or four autospores were observed. Moreover, under the CR condition, following Ca^2+^ replenishment, a gradual increase in n cell population was observed (Fig. 2B).

### Transcriptome Analysis of *C. sorokiniana* Strain DSCG150 according to Presence/Absence of Ca^2+^

RNA sequencing was conducted to observe gene expression in DSCG150 in the presence and absence of Ca^2+^ in the medium with samples at early log-phase (32 h) cultivated under NG and CA conditions. The transcriptomes obtained by RNA-Seq. were annotated using blastx, and a total of 53 genes associated with the cell cycle were identified. The genes were categorized as follows: DNA replication/damage (seven genes), transcription factors (two genes), cell division cycle proteins (16 genes), cyclins/ cyclin- dependent kinases (11 genes), anaphase-promoting (nine genes), calcium- dependent proteins (one gene), mitogen-activated protein kinases (five genes), and histone-lysine N- methyltransferase (two genes).

**Fig. 3:**
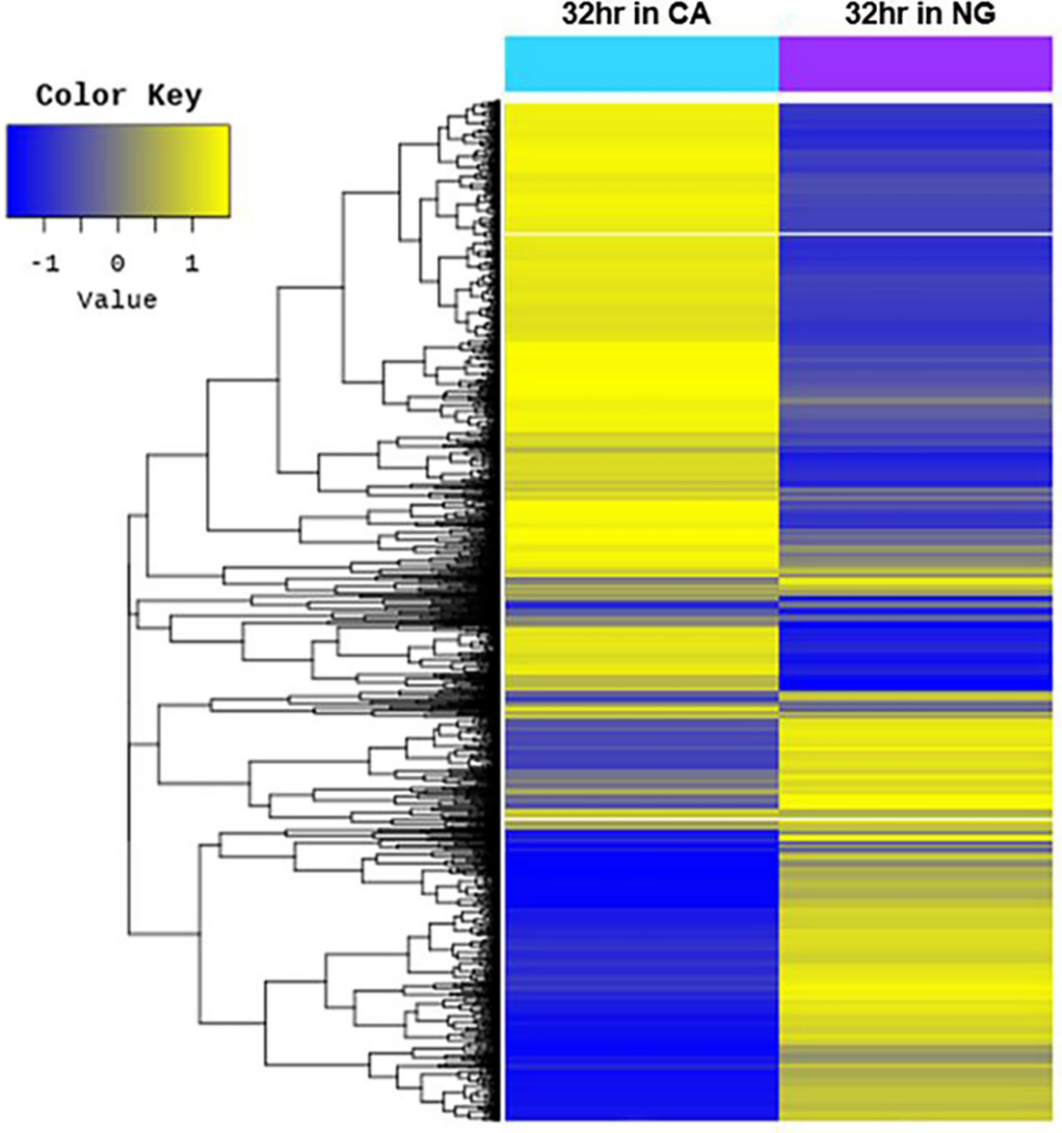
Heat map of genes differentially expressed of *C. sorokiniana* strain DSCG150 during cultivation in the medium with 0.5 mM Ca^2+^ (NG) and absence of Ca^2+^ (CA). DEG analysis was conducted using cells cultivated for 32 h in CA and NG condition. The heat map shows a visualization of grouped information for each sample and gene based on the similarity of expression patterns using hierarchical clustering (distance metric= Euclidean distance, linkage method= complete) analysis with Z-score for normalized value (log2 based) for significant genes. To identify genes exhibiting significant expression changes under CA condition, we compared the DEGs to the cells grown under NG condition. A total of 2,353 genes were expressed differently, among them 1,369 genes were upregulated and 984 genes were downregulated.

To evaluate the differences in gene expression of DSCG150 cells cultivated under NG and CA conditions, DEGs analysis was performed with the same samples (at 32 h). DEG analysis revealed that a total of 2,353 genes were expressed differentially depending on the presence and absence of Ca^2+^. Compared to the NG condition, 1,369 genes were upregulated and 984 genes were downregulated under CA condition (Fig. 3).

Among the 53 genes related to the cell cycle analyzed above, 4 genes; Dual specificity protein phosphatase CDC14A (*CDC14A*), origin of replication complex subunit 6 (*ORC6*), calmodulin (*calA*), and cell division control protein 2 (*CDC2_1*), exhibited down- or up- regulation exceeding two-fold in the DEGs analysis (Table 2). *CDC14A* and *ORC6* genes, with a 2.32 and 2.01-fold decrease in expression, respectively, were selected. Additionally, *calA* and *CDC2_1* genes, exhibiting a 5.05-fold and 33.27-fold increase, respectively, were also selected.

**Fig. 4:**
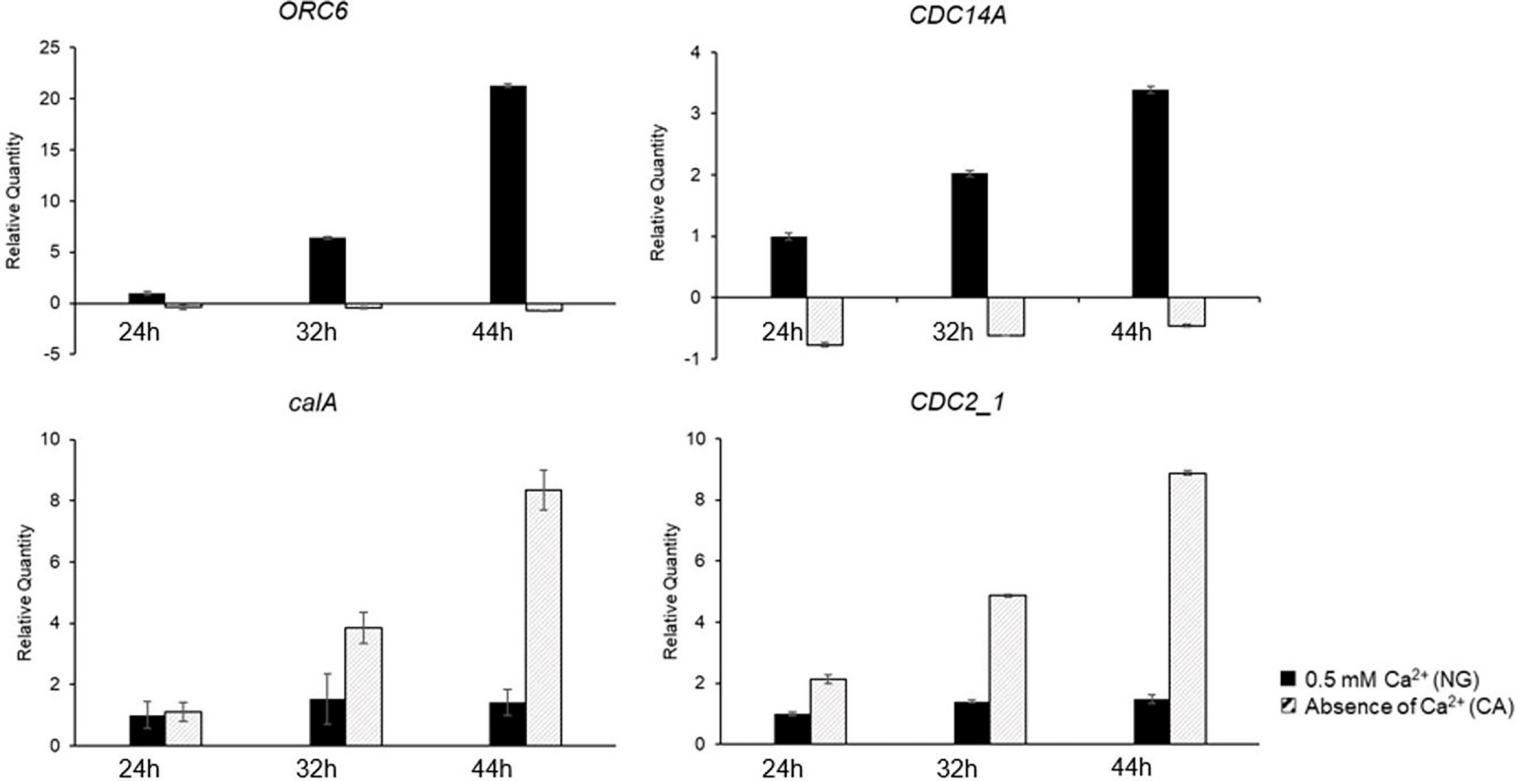
Time-course expression patterns of selected differentially expressed genes from *C. sorokiniana* strain DSCG150 cultivated in the medium 0.5 mM Ca^2+^ (NG) and absence of Ca^2+^ (CA). The transcript levels were calculated relative actin. All data were normalized to the transcript levels in corresponding genes of the cell grown for 24 h under the NG condition. Cells in the lag-phase for 24 h; 32 h in the early log-phase; 44 h, in the log-phase. The growth phase was defined based on the growth curve in NG condition due to no logarithmic growth under the CA condition. The values are shown as mean ±SD from three independent replicates.

In both NG and CA conditions, at the lag (24 h), early log (32 h), and log phase (44 h), the expression patterns of the four selected genes were analyzed using qRT-PCR (Fig. 4). Under NG conditions, the expression levels of the *CDC14A* and *ORC6* genes were increased by 2.02- and 6.38-fold, respectively, at 32 h, and by 3.39- and 21.31-fold, respectively, at 44 h, compared to that at 24 h. In contrast, under CA conditions, there were no notable changes in expression over the cultivation period. Under NG condition, *calA* and *CDC2_1* did not show significant expression differences during cultivation (Fig. 4). Under CA conditions, the expression level of the *calA* gene was similar to that under NG conditions at 24 h, however, at 32 and 44 h, expression of *calA* increased 3.85× and 8.35×, respectively. Additionally, the expression of *CDC2_1* increased 2.13× (24 h), 4.87× (32 h), and 8.88× (44 h).

## Discussion

*C. sorokiniana* strain DSCG150 showed growth in the early stage of incubation, regardless of the presence/absence of Ca^2+^ supply, but over time, the cells exhibited logarithmic growth under the NG condition and growth stagnation under the CA condition. Microalgal cell division involves the production of two to eight autospores in a single cell by repeated regular cell cycle (G–S–M phases). Subsequently, daughter cells (n) are produced by fission and the cell cycle is repeated [21].

In the flow cytometry analysis, we found that DSCG150 produced n to 8n autospores.

Under the NG condition, 2, 4, and 8n autospores were all present, but a population consisting mostly of n-type daughter cells was maintained. Under normal cell cycle, G_1_ to S is the longest phase of the cell cycle [21], thus, the cell population will consist primarily of n daughter cells. Therefore, the simultaneous occurrence of n cells with two- 8n cells suggests the occurrence of a normal cell cycle.

Confocal and flow cytometry analyses performed under the CA condition showed that at 32h, the proportion of autospore cells (2n, 4n) increased, while 4n autospores became more dominant over time (Fig. 2A). Confocal image analysis results showed that 4n autospores were separated from each other (Fig. 2B), suggesting that *Chlorella* cells underwent mitosis and cytokinesis under CA conditions [21], but the process of fission from 4n autospores to n daughter cells was inhibited, which inhibited the cell proliferation process (Fig. 1A).

DEG analysis was performed to investigate the differences in gene expression of *Chlorella* cells cultivated under NG and CA conditions. The majority of cell cycle- associated genes were expressed regardless of the presence or absence of Ca^2+^, with over 90% of the genes (49 out of 53) showing less than a two-fold change in expression. Of these, four genes (*calA*, *CDC2_1*, *CDC14A*, and *ORC6*) exhibited a fold change of >2. Under the CA condition, progression of the cell cycle up to 4n autospores was observed. Accordingly, the effects of the four genes that showed differences in expression according to the presence/absence of Ca^2+^ on the cell cycle were analyzed by qRT-PCR.

ORC is a protein that binds to the replication origin to initiate DNA replication, which acts together with Cdc6, Cdt1, and Mcm DNA helicase to form a prereplicative complex for DNA replication initiation [23]. In the human *ORC6* model (HeLa cell), deletion of the *ORC6* gene caused reduced DNA replication, formation of multipolar spindles, aberrant mitosis, and phenotype change, such as the formation of multinucleated cells. In a *ORC6* mutation study using *Drosophila* cells, deletion of the *ORC6* gene caused cell cycle arrest at G_1_ and M phases [23-25]. In this study, the *ORC6* gene was upregulated under the NG condition, while the expression level increased gradually along with logarithmic cell growth. It is speculated that the increase in the expression of the *ORC6* gene under NG condition could be attributed to vegetative cells with multiple autospores including n cells exhibite active fission during the cell cycle. Under CA condition, *Chlorella* cells showed accumulation of 4n autospores that cannot undergo fission, and as a result, no further DNA replication took place and the expression of *ORC6* gene was maintained at a low level.

CDC14 is a protein with dual specificity protein phosphatase activity in the dephosphorylation of threonine and tyrosine residues. In a budding yeast model, CDC14 dephosphorylates cyclin dependent kinase (Cdk) to promote mitotic exit and cytokinesis, the final events of a cell cycle. In the human U-2_OS cell line, CDC14A regulates centrosome separation and duplication during mitosis [26-28]. In our study, the *CDC14A* gene was upregulated under the NG condition and the expression level increased with further growth. It is believed that the increase in the expression of the *CDC14A* gene under the NG condition could be attributed to the high proportion of n cells that can easily undergo fission (mitotic exit and cytokinesis activation), whereas under CA condition with the accumulation of 4n autospores, fission was not possible, and thus, the expression of this gene was maintained at a low level.

**Fig. 5:**
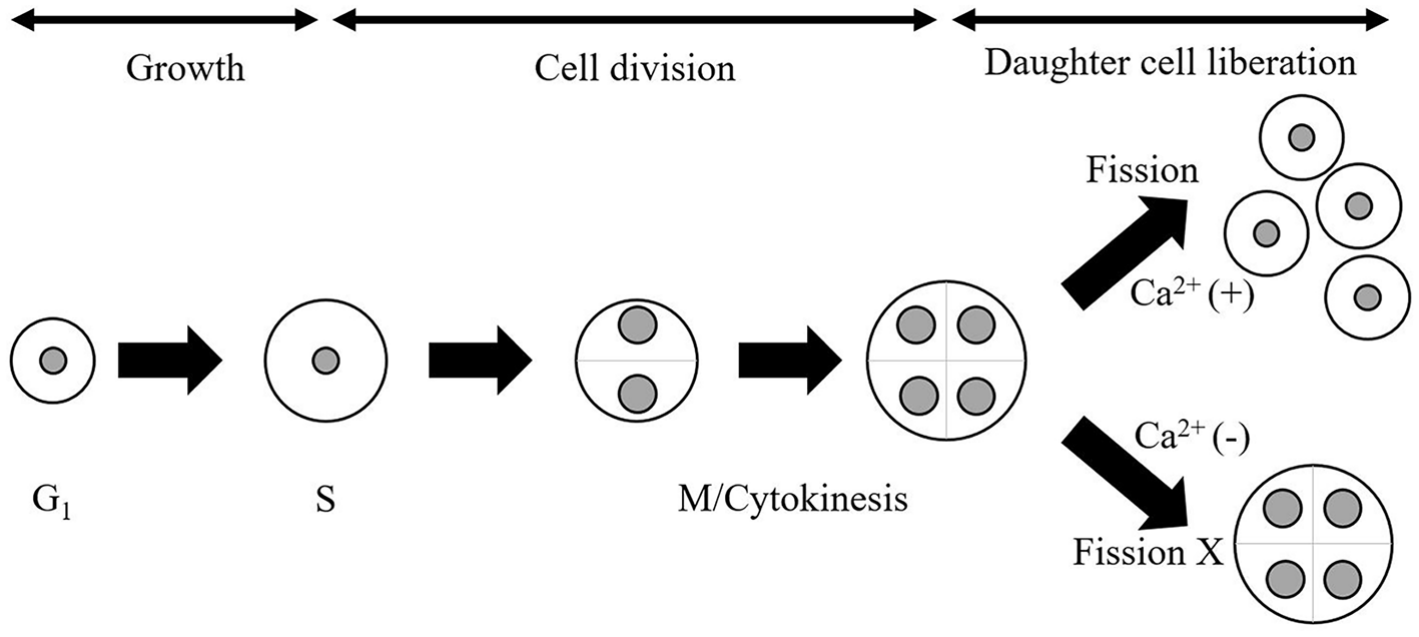
Growth cycle of *C. sorokiniana* strain DSCG150 including multiple fission model. The strain *C. sorokiniana* strain DSCG150 exhibits growth through a multiple fission model. Autospores divided passing through a cell cycle (G1, S, and M/cytokinesis) in a single cell are liberated to daughter cells through a final event, ‘**fission process**’. We defined the entire process, including the fission process as, ‘**growth cycle**’. The daughter cells separated through the growth cycle subsequently undergo a new growth cycle.

Calmodulin is a primary intercellular receptor that plays an important role in cell cycle regulation. Increased calmodulin expression shortens the G_1_ phase to accelerate the cell cycle, whereas decreased calmodulin expression causes cell arrest in G_1_/S, G_2_/M, and metaphase/anaphase transition [29]. In our study, the *calA* gene was downregulated under the NG condition and expression was maintained at a consistent level during cultivation. It is believed that under the NG condition, repeated growth cycles (Fig. 5) are carried out well, and as a result, expression was maintained at a consistent level throughout the cultivation. Under CA conditions, *calA* was upregulated and the expression level increased as cultivation time elapsed. The increase in *calA* expression under CA conditions may be attributed to the accumulation of 4n cells increasing expression to accelerate the cell cycle to generate n cells.

Cdk1 (known as a CDC2 homolog) triggers polarization of actin cytoskeleton and bud emergence in late G_1_ phase in yeast model [30]. Under CA conditions, the gene expression increased as the cultivation progressed. It is speculated that the expression of *CDC2_1* increased according to the increase in the 4n autospore population under CA condition. However, the n *Chlorella* population was predominantly found under the NG condition, and as a result, expression of this gene was maintained at a consistent level.

With the expression patterns of these four genes, n cells under NG condition enhanced DNA replication and protein phosphatase activity to carry out the growth cycle, including fission. However, 4n cells under the CA condition enhanced the expression of genes to accelerate the cell cycle for transition to n cells.

When *Chlorella* cells with a stagnant growth cycle due to the absence of Ca^2+^ were replenished with Ca^2+^ (CR condition), n cells increased and growth was restored (Fig. 2A), suggesting that Ca^2+^ is critical in the fission process of the *Chlorella* growth cycle.

In conclusion, Ca^2+^ affects the growth cycle of *C. sorokiniana* strain DSCG150 and plays a key role in the fission process. Absence of Ca^2+^ inhibited the growth cycle of *Chlorella* by promoting 4n cell accumulation and inhibiting fission into n cells. The findings also showed that 4n cells with an inhibited growth cycle were able to transition to n cells and restore growth after Ca^2+^ replenishment. Therefore, Ca^2+^ is a key metallic element in the cell growth cycle and is directly involved in the fission of *Chlorella*.

## Supplemental Materials

Supplementary data for this paper are available on-line only at http://jmb.or.kr.



## Figures and Tables

**Table 1:** Growth performance of *C. sorokiniana* strain DSCG150 in the medium with 0.5mM Ca^2+^ (NG), absence of Ca^2+^ (CA) and additional 0.5 mM Ca^2+^ (CR).

Culture condition	Final Cell No., × 10^8^ cells/ml	Specific growth rate, /h	Final residual glucose in medium, %	Ca^2+^ consumption for cell growth, mg/l
NG	5.01 ± 0.08	0.0735 ± 0.001	N.D.	4.57 ± 0.006^2)^
CA	0.61 ± 0.01	0.0248 ± 0.001	1.23 ± 0.002	N. D.
CR	4.90 ± 0.05	0.0508 ± 0.002^1)^	N.D.	7.61 ± 0.006^3)^

Additional 0.5 mM Ca^2+^ was inoculated after 48 h to culture broth cultivated in CA condition. The values are shown as mean ±SD from three independent replicates.

^1)^The specific growth rate was calculated based on the growth data since the addition of 0.5 mM Ca^2+^.

^2)^The amount of Ca^2+^ consumed for 48 h.

^3)^The amount of Ca^2+^ consumed for 36 h after addition of Ca^2+^.

**Table 2:** Expression differences of genes related to cell cycle of *C. sorokiniana* strain DSCG150 cultivated in medium with 0.5 mM Ca^2+^ (NG) and absence of Ca^2+^ (CA).

Gene ID	Gene symbol	Greatest identity, %	Lowest E- value	Gene description	Fold-changes
LOCUS_008256	*calA*	92.81	9.41 × 10^-85^	Calmodulin	5.05
LOCUS_004852	*CDC2_1*	86.27	0	Cell division control protein 2	33.27
LOCUS_002856	*CDC14A*	92.07	0	Dual specificity protein phosphatase CDC14A	-2.32
LOCUS_005832	*ORC6*	87.67	1.84 × 10^-130^	Origin of replication complex subunit 6	-2.01

RNA- sequencing was conducted on cells at 32 h under NG and CA condition. Four genes exhibited up- or downregulation according to the differentially expressed genes (DEGs) data compared to the NG condition.
