# Supplementary material for: The Impact of Calcium Depletion on Proliferation of Chlorella sorokiniana Strain DSCG150
Source: J Microbiol Biotechnol. 2024 May 29;34(7):1425–32. doi: 10.4014/jmb.2403.03018 (PMC11294638; doi:10.4014/jmb.2403.03018)
Supplement: Supplementary file 1 [file jmb-34-7-1425-supple.pdf]

J. Microbiol. Biotechnol. 2024. 34(7): 1425–1432

https://doi.org/10.4014/jmb.2403.03018

Supplementary Figures and Table

The Impact of Calcium Depletion on Proliferation of *Chlorella sorokiniana* Strain DSCG150

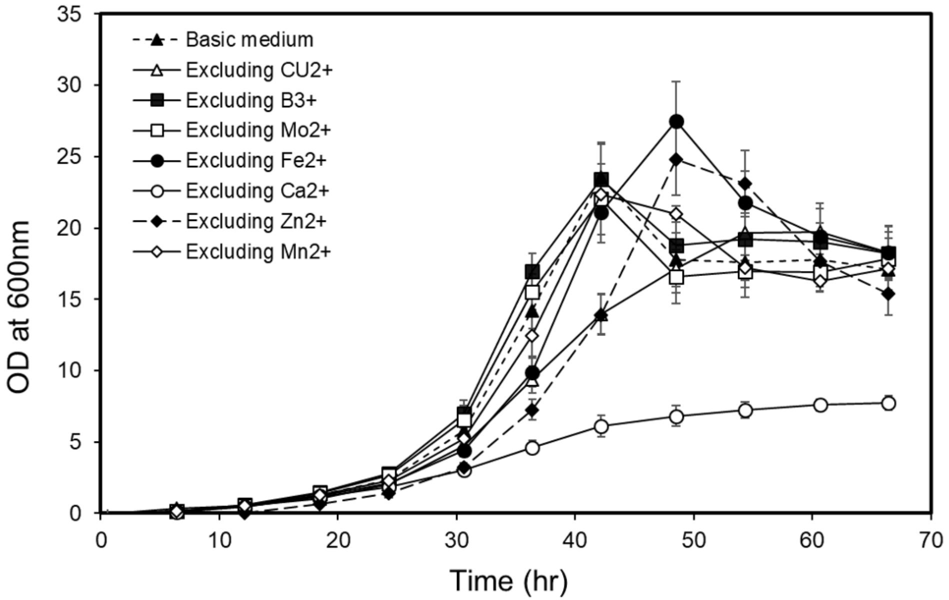

Fig. S1. Effects of elimination of metal ion source on growth of *C. sorokiniana* strain DSCG150. The basic medium used was Sanawa and Endo medium IV and individual metallic elements were excluded from the medium (except Mg^{2+}). Particularly, it was observed that exponential growth was not observed, and growth reached stagnant when Ca^{2+} was omitted from the medium. The BIOSAN RTS-8 personal multi- channel bioreactor (BIOSAN, Latvia) was used for the cultivation, with a volume of 20 mL, using a 5-day cultured plate colony as the seed, and the

inoculum was standardized to an initial OD at 600nm of 0.6. Cells were grown at 30℃, 2700 RPM supporting k_La of 450/h, and OD at 600 measurements every 6 hour were automatically conducted for growth- monitoring. The values are shown as mean ±SD from three independent replicates.

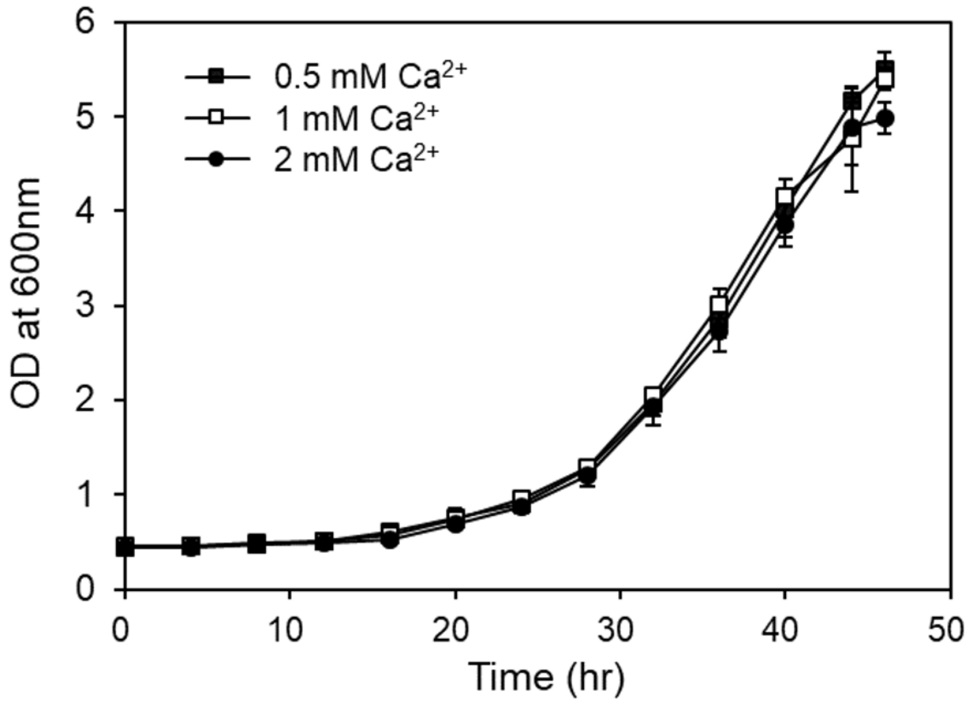

Fig. S2. Growth of *C. sorokiniana* DSCG150 on different Ca^{2+} concentration. *C. sorokiniana* strain DSCG150 was cultivated in 1 L Erlenmeyer baffled flasks containing 250 mL modified Sanawa et. al. [12] medium in which varied with Ca^{2+} concentration of 0.5, 1, and 2 mM. Cells were cultivated at 30°C shaking incubator with 140 RPM in dark conditions; 1 mL of 5-day-old cells on agar plate were used for the inoculum and the initial optical density at 600nm for each was 0.4–0.45. Samples were taken every 4 h and OD at 600nm was determined. The values are shown as mean ±SD from three independent replicates.

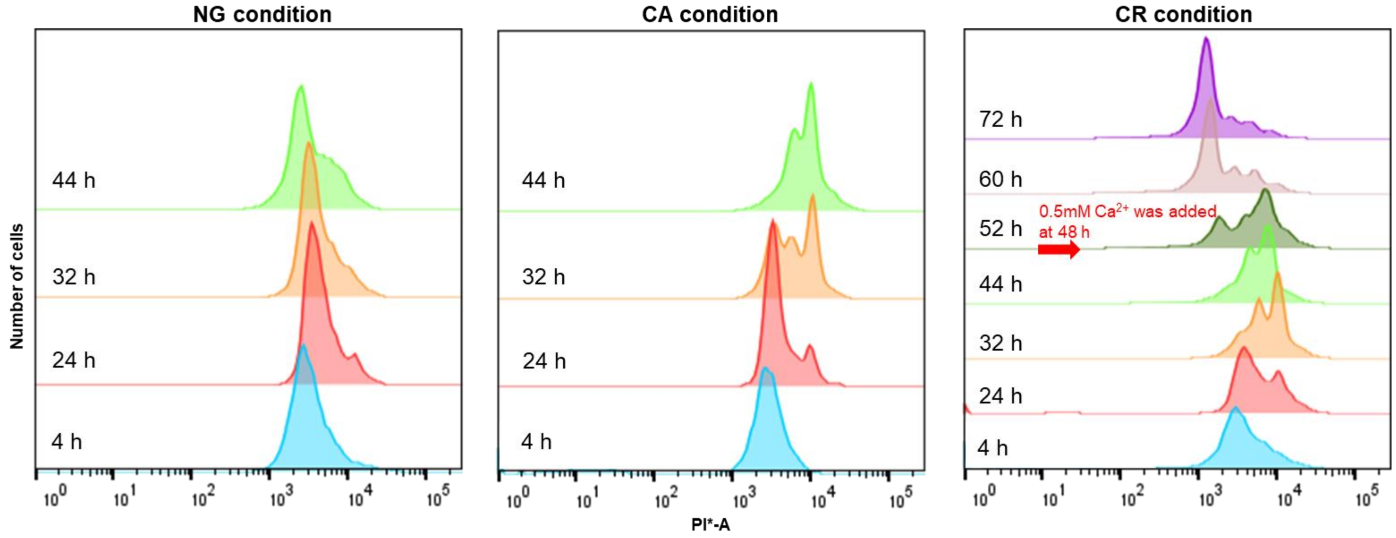

Fig. S3. Overlap of the fluorescence histograms of cells grown in the medium with 0.5 mM Ca^{2+} (NG), absence of Ca^{2+} (CA), and additional 0.5 mM Ca^{2+} (CR). The red arrow represents a point to supply the additional 0.5 mM Ca^{2+} to culture broth of CA condition. Cells from each cultivation time were thermal fixed and stained by PI dye for the flow cytometry analysis. X- and y-axes represent fluorescence intensity and cell number, respectively.

Table S1. Selected genes and primer pairs used for qRT-PCR.

Target gene symbol	Gene description	Primer sequences	Product size (bp)
calA	Calmodulin	5’-TAGCCTGTTTGACACCGACC<br>3’-CAAACTGCAGCACATACGCC	179
CDC2_1	Cell division control protein 2	5’-GTCAGCAGGGGTGCTGTATT<br>3’-TATGTTGTGCGGCTTCAGGT	187
CDC14A	Dual specificity protein phosphatase CDC14A	5’-AGGTGGGCTTCATCAACTGG<br>3’-GCTCCCGGAAGTACTCGATG	201
ORC6	Origin of replication complex subunit 6	5’-GCCTACAAGGAGCGATTCGT<br>3’-TCACCTGGGCAAACTCAGTG	181
